# Factor structure of post-operative quality of recovery questionnaire (QoR-15): An Italian adaptation and validation

**DOI:** 10.3389/fpsyg.2022.1096579

**Published:** 2023-02-01

**Authors:** Rosalba Rosato, Valentina Palazzo, Felice Borghi, Marco Camanni, Andrea Puppo, Elena Maria Delpiano, Luca Pellegrino, Elisa Piovano, Alessio Rizzo, Monica Rolfo, Mario Morino, Marco Ettore Allaix, Silvia Testa, Giovannino Ciccone, Eva Pagano

**Affiliations:** ^1^Department of Psychology, University of Turin, Turin, Italy; ^2^Clinical Epidemiology Unit, Città della Salute e della Scienza Hospital, Torino and CPO Piemonte, Turin, Italy; ^3^Department of Surgery, University of Torino, Turin, Italy; ^4^Oncological Surgery, Candiolo Cancer Institute-FPO-IRCCS, Turin, Italy; ^5^Obstetrics and Gynecology Unit, Martini Hospital – ASL Città di Torino, Turin, Italy; ^6^Obstetrics and Gynecology Unit, Santa Croce e Carle Hospital, Cuneo, Italy; ^7^Obstetrics and Gynecology Unit 3, AOU Città della Salute e della Scienza di Torino, Turin, Italy; ^8^Ordine Mauriziano Hospital, Turin, Italy; ^9^Healthcare Services Direction, Humanitas, Turin, Italy; ^10^Department of Surgical Sciences, University of Turin, Turin, Italy; ^11^Department of Human and Social Sciences, University of Aosta Valley, Aosta, Italy

**Keywords:** quality of recovery, psychometric validation, colorectal surgery, hysterectomy, exploratory structural equation modelling

## Abstract

**Background:**

The Quality of Recovery questionnaire (QoR-15) is an English instrument for measuring quality of recovery in surgical patients, not yet translated and validated in Italian when the Enhanced Recovery After Surgery (ERAS) Piemonte studies were planned.

**Objective:**

To produce the Italian version of the QoR-15 questionnaire, to evaluate its factorial structure and to assess the invariance between two types of surgery.

**Methods:**

The Italian version (QoR-15I) was obtained translating and adapting the original version to the Italian context. The validation was performed suppling the QoR-15I to 3,784 patients enrolled in two parallel stepped wedge cluster randomised trials (ERAS Colon-rectum Piemonte; ERAS Gyneco Piemonte). The factor structure and its invariance between types of surgery was tested using confirmatory bifactor model and multi-group analysis. Comparative fit index (CFI), root mean square error of approximation (RMSEA), and standardized root mean square residual (SRMR) fit indices and their changes between nested models were used to assess the factor structure and the invariance.

**Results:**

The bifactor model showed good fit (RMSEA = 0.049, CFI =0.957, SRMR = 0.036) and provided a general recovery factor and two specific factors for physical and mental recovery. Eighty-four percent of the common variance is attributable to the general factor, and thus the QoR-15I is sufficiently ‘one-dimensional’ with an adequate reliability (ω_h_ = 0.70). The ωs values for the physical and mental recovery factors were 0.01 and 0.13, respectively. Multigroup analysis supported configural (RMSEA = 0.053, CFI = 0.950, SRMR = 0.035) and metric invariance (ΔRMSEA = -0.004; ΔCFI = -0.002; ΔSRMR = 0.014), whereas the intercept constraint was removed from item 15 to obtain partial scalar invariance (ΔRMSEA = 0.002; ΔCFI = 0.007; ΔSRMR = 0.004). Construct validity was supported by a negative association of QoR-15I scores with all variables related to worse patient condition and more complex surgery.

**Conclusion:**

Our results support the use of the QoR-15I as a valid, reliable, and clinically feasible tool for measuring the quality of recovery after surgery. The results of the confirmatory factor analyses suggest that a unique recovery score can be calculated and support measurement invariance of the QOR-15I across the two type of surgery, suggesting that the questionnaire has the same meaning and the same measurement parameters in colorectal and gynaecologic patients.

## Introduction

Enhanced recovery after surgery (ERAS) is a structured perioperative care pathway with multiple evidence-based interventions aimed at reducing surgical stress and accelerating recovery. Many studies have assessed recovery primarily using clinical measures such as length of hospital stay, postoperative complications, and readmission rates, which do not account for the complexity of the recovery process and do not capture the patient’s perspective. Patient-reported outcomes have been recognized as an important component of a set of outcomes appropriate for evaluating the effectiveness of an ERAS program (Feldman et al., 2015).

Postoperative recovery is a complex process influenced by various factors such as the patient’s health status, anaesthesia, and surgical techniques. These factors may be associated with many sequelae, such as the occurrence of complications, morbidity, and mortality. A very important aspect in the assessment of postoperative recovery is the quality of this recovery by the patients, in order to also assess the patients’ well-being, the general quality of life and the quality of recovery after anaesthesia. One of the most widely used questionnaires is the Quality of Recovery-40 (QoR-40) questionnaire, which was developed by Myles in 2000 as an instrument to measure quality of recovery from the patient’s perspective (Myles, 2020). It is composed of five dimensions (pain, physical comfort, physical independence, emotional status and psychological support) theoretically grouped into a physical well-being domain (pain, physical comfort and physical independence) and a mental well-being domain (emotional status and psychological support) (Stark et al., 2013; Kim et al., 2020). This two dimensional structure has never been empirically tested. Later, a 15-questions version (QoR-15) was developed based on a literature review and consultation with experienced anaesthesia and research nurses. The QoR-15 has good validity, reliability, responsiveness, clinical acceptability, and feasibility of the score, with most patients able to complete the questionnaire in less than 3 min (Stark et al., 2013; Myles et al., 2022).

The short version, consisting of 15 questions, includes questions on each of the five dimensions included in the original QoR-40 questionnaire. Although the selected questions are from different dimensions of recovery, the QOR-15 has always been used and validated as a unidimensional instrument to measure the quality of recovery, with the highest score indicating the best recovery, and the two-dimensional structure indicated by Stark and colleagues (Stark et al., 2013) has not been empirically tested. To our knowledge, few studies have examined the factor structure of the QOR-15 using exploratory factor analysis (Kim et al., 2020; Myles, 2021). Kim and colleagues in 2020 (Kim et al., 2020) investigated the structural validity of the Korean QoR-15 using exploratory factor analysis, which showed acceptable results, but they did not discuss the solution they found. They calculated the physical and mental components of recovery following Stark’s original specification and used it to assess convergent validity with the physical and mental composite scores of the SF-36 questionnaire. In addition, exploratory factor analysis conducted by Myles (2021) revealed an essentially unidimensional solution, supported by the fact that the first factor accounted for 30.9% of the total variance and the ratio of factor 1 to factor 2 was 3.37. A single study examined the factor structure in a confirmatory approach by applying a bifactor model to the data from some of the studies included in a meta-analysis (Kleif et al., 2018). In this case, the results also supported the essential unidimensionality of the QoR-15.

The QoR-15 has already been translated and validated in several languages (Kleif et al., 2015; Sá et al., 2015; Bu et al., 2016; Lyckner et al., 2018; Demumieux et al., 2020; Lee et al., 2021), but the Italian translation and validation was not yet available at the time we designed the study.

As part of the EASY-NET^1^ project, which aims to evaluate the effectiveness of an Audit&Feedback approach in improving quality of care, we conducted two parallel, stepped wedge cluster randomised trials to implement the ERAS program throughout the hospital network of the Piemonte region (4.3 million inhabitants in northwestern Italy). One study focused on colorectal cancer surgery (ERAS Colon-rectum Piemonte) (Pagano et al., 2021) and the other on hysterectomy for both malignant and benignant diseases (ERAS-Gyneco Piemonte) (Piovano et al., 2022). The aim of both studies was to estimate the true impact of the ERAS protocol on a large, unselected population. To measure quality of recovery after surgery, the QoR-15 questionnaire was identified as an appropriate instrument.

The objective of the present study is to describe the translation, adaptation, and validation of the QoR-15 questionnaire for the Italian context. As part of the validation process, we also intended to assess the factor structure of the QoR-15I and the measurement invariance between the two types of surgery. Factorial validity is one way to provide support for construct validity; construct validity is important for making inferences from scale scores about the underlying construct of interest. We examined measurement invariance (i.e., equivalence) across subgroups because patients underwent to different type of surgery and severity factors might influence perceptions of QoR (i.e., colorectal vs. gynecological patients, or malignant vs. benign cancer).

## Methods

### Data collection

The psychometric evaluation was performed using data collected in two large pragmatic clinical trials, the ERAS Piemonte studies. Details of the study protocols have been published previously (Pagano et al., 2021; Piovano et al., 2022). Only patients with very high complexity or clinical severity (mainly, ASA V), to be recorded on the study Case Report Form (CRF) at the time of enrollment, were excluded. The length of hospital stay was the primary outcome and postoperative complications, quality of recovery, 30-day readmissions, patient satisfaction, and healthcare costs were the secondary outcomes. Study protocols were approved by the Ethics committees of all participating units. Each patient signed an informed consent form at the time of enrollment in the study.

Participating patients were asked to complete the QoR-15I questionnaire 48 (up to 72)-hours after colorectal surgery and 24 (up to 48)-hours after hysterectomy; time of administration was different because of the different severity of clinical conditions after surgery in the two groups. The questionnaire contained 15 questions and the scale visual analogue scale (VAS), in which patients were asked to rate their current health status on a scale from 0 (worst health status) to 10 (best health status). Nurses and obstetricians were required to give patients the paper questionnaire, monitor completion, and provide assistance as needed.

### Measures

#### QoR-15I questionnaire

The QoR-15 consists of 15 items rated on an 11-point rating scale ranging from 0 to 10. The overall QoR-15 score was obtained from the sum of all items, ranging from 0 to 150, where higher scores indicate a better quality of recovery.

The instrument was translated using a forward and backward translation method (Guillemin et al., 1993). First, the original QoR-15 was independently translated from English into Italian by two native Italian speakers with excellent knowledge of English and experience with health care terminology. They met to compare their translations and agreed on discrepancies to produce a preliminary Italian version. This was then blindly back-translated into English by a native English lecturer (GW). A final Italian version (QoR-15I) was produced by the authors (GC, EP, RR) after discussion and comparison between the original, the preliminary, and the back translation. Inconsistencies were identified and corrected.

A cognitive debriefing was then conducted with a sample of 17 patients, who were asked to read and review the translated version to identify unclear words or concepts. The results of the debriefing were used to revise the final version.

#### General VAS health status

A visual analogue scale (VAS) was employed to assess patients’ current health status on a scale from 0 (worst health status) to 10 (best health status).

#### Sociodemographic and clinical information

The following variables were considered for the validation of the Italian version of QOR-15: gender, age, education, employment, marital status, the subjective assessment of the patient’s overall health by the American Society of Anesthesiologists (ASA) score, the presence of comorbities through the Charlson Comorbidity Index, surgery techniques (open or minimally invasive), length of hospital stay, intensive care unit (ICU) stay, incidence of post-operative complications. The performance of a stoma in colorectal cancer patients and a diagnosis of malignancy in gynecologic patients were also considered in the analyses.

### Statistical analysis

Mean and standard deviation were used to describe normally distributed variables, median and interquartile range for skewed variables, and frequencies and percentages for categorical variables. Correlations were measured with Pearson’s r coefficients. The t test was used to compare two means. Hedge’s g effect size was calculated as the standardized mean difference in QoR-15I values.

### Psychometric evaluation

#### Factor structure

Given the dual purpose of the work to examine the factor structure of the QOR-15I and to test the measurement invariance by type of surgery, the sample was randomly divided into two sub-samples using the odds and evens split method. One half of the sample (test group n = 1892) was used to identify the most appropriate factor model, examining the goodness of fit and the size of the loadings of alternative confirmative factor analysis (CFA) models. The second half of the sample (validation group, n = 1892) was used to cross-validate the factor structure obtained in the test group and to assess the measurement invariance across the two types of surgery.

In the test group, we first examined the goodness of fit of the one-factor model, in which all 15 items where indicators of a latent dimension of quality of recovery, and of the theoretical two-factor model in which items of physical comfort (item 1–4, 13), physical independence (item 5, 8) and pain (item 11–12) were indicators of the physical dimension of the quality of recovery and items of emotional state (item 9–10, 14–15) and psychological support (item 6–7) were indicators of the mental dimension of the quality of recovery. The two-factor model was estimated twice, once constraining to zero the covariation between the two factors and then allowing them to be correlated. Because of the unsatisfactory results of these initial analyzes, we then applied the Exploratory structural equation modeling approach (ESEM) to find a correlated two-factor solution that provided a better fit to the data. In the light of the ESEM results, a bifactor model was then estimated. The bifactor model represents a plausible and useful alternative to the higher-order models traditionally used to maintain a unidimensional structure consistent with the theoretical basis on which the measure was constructed, while also taking into account important item grouping factors (Reise et al., 2013; Kleif et al., 2018). Because the general and group factors are uncorrelated in a bifactor model, there is little point in constructing subscales when factor loadings are high on the general factor and low on the group factors. Items with a loading ≥of 0.30 on general and group factors are acceptable indicators of the latent dimension being measured (Brown, 2015).

To assess the relative strength of the general quality of recovery factor compared to the group factors, the explained common variance (ECV) and omega hierarchical (ω_h_) were calculated. Omega for each group factor (ω_s_) was calculated to assess the extent to which the subscale scores are reliable measures of the corresponding specific latent variables once the items’ joint variance due to the general factor was removed (Reise et al., 2013). In all factor models, 5 error covariances between items were estimated: 2 error covariances due to the wording in the Italian version (Q3/Q4, Q14/Q15) and 3 error covariances between pairs of items that were the only 2 items covering one of the five dimensions of the original QoR40 (Q5/Q8, Q6/Q7, and Q11/Q12). We considered the model fit acceptable when the following criteria were met: Root Mean Square Error of Approximation (RMSEA) < 0.08; Comparative Fit Index (CFI) > 0.90; and Standardized Root Mean Square Residual (SRMR) < 0.08 (Hu et al., 1995; Hu and Bentler, 1999).

#### Measurement invariance

The measurement invariance between the two types of surgery (colon cancer surgery vs. hysterectomy) was investigated in the independent validation group (Horn and McArdle, 1992) using a multi-group CFA framework. The best fitting model from the test group was estimated on the entire validation group and then separately on patients who underwent to colorectal or gynecological surgery to ensure that the same measurement model was supported in each group. We tested three increasingly restricted levels of measurement invariance. First, we analyzed configural invariance to check whether the same pattern of loadings was present in the two surgical samples: a lack of configural invariance suggests that the observed items measure different constructs in the two groups. Second, we tested metric invariance, which assumes that the factor loadings are the same in all groups. When factor loadings are invariant across groups, it is assumed that measurements are on the same scale across groups and that the underlying latent factors are measured in the same way across groups. Finally, we tested scalar invariance, which also requires the assumption that the item intercepts are invariant across groups (Millsap and Yun-Tein, 2004). If item intercepts are not invariant across groups, this suggests that participants in at least one of the groups tend to answer systematically higher or lower, even when factor loadings are invariant across groups. According to Chen (2007), different cutoff points are used for testing invariance at different levels, as SRMR seems to be more sensitive to lack of invariance in factor loadings than in intercepts or residual variances, while CFI and RMSEA seem to be equally sensitive to all 3 types of lack of invariance. When tested for metric invariance, a change of ≥0.010 in CFI (ΔCFI) supplemented by a change of ≥0.015 in RMSEA (ΔRMSEA) or a change of ≥0.030 in SRMR (ΔSRMR) is indicative of a lack of invariance. When testing for scalar invariance, the cutoff value for SRMR is 0.010, whereas it does not change for the other two indices.

All factor models were estimated with the maximum likelihood estimation with robust standard errors (MLR).

#### Reliability and validity

Based on the results from the factor structure analysis, and according to the literature, a single QOR score was calculated as the sum of the 15 individual items. All reliability and validity analyses were conducted using the entire data sample (n = 3,874). Reliability was tested by internal consistency Cronbach’s alpha and split-half (Cohen, 1988) and by the McDonald’s coefficient omega total (ω_t_), a model-based estimate of reliability obtained from the bifactor solution on the entire sample, considering all sources of common variance (general and group factor) (Reise et al., 2013). We considered values in the range of 0.70 and 0.90 as measures of good internal consistency and reliability (Nunnally and Bernstein, 1994).

Construct validity was tested using the hypothesis that a negative association would exist between the QoR-15I score and some variables at baseline (high ASA score, Charlson comorbidity index ≥1) or during hospitalization (duration and type of surgical approach, open or minimally invasive) and two study outcomes (length of hospital stay and occurrence of medical or surgical complications). We also identified two proxies of disease severity: presence of stomia in colorectal cancer and malignant/benign disease for hysterectomy. We hypothesized laparoscopic surgery, absence of stomia and benign diseases to be associated with a higher QoR-15I score. A positive association between QoR-15I scores and well-being VAS was also expected. A gender comparison was only possible for colorectal cancer, and we assumed lower QoR-15I scores for women, as the female gender is known to have poorer postoperative recovery than men (Buchanan et al., 2011).

The feasibility of the QoR-15I was assessed in terms of recruitment and completion rates.

The ceiling or floor effect was considered to be present if at least 15% of the responses achieved the highest or lowest possible response value, respectively (Terwee et al., 2007). Statistical analyses were performed in SAS (SAS Institute, 2017); CFA and invariance analyses were performed in MPLUS 8.0 (Muthén and Muthén, 1998). A value of *p* <0.05 was considered statistically significant.

## Results

The final Italian version of the QoR-15, named QoR-15I, is reported in the (Supplementary material; Supplementary Table S1). During cognitive debriefing, all questions were found to be clear and understandable. Question 4 “Have had a good sleep” was translated as “Have had sleep” because most patients complained of difficulty sleeping in the hospital; question 8 “Return to work or usual home activities” was reduced to “Return to usual activities” because in colorectal cancer surgery not only the prognosis requires a long absence from work (about 4 weeks), but also many patients are likely to be retired because of their advanced age. No other cultural adjustments were made.

Patients enrolled in the two ERAS Piemonte studies were 5,226 (2,923 in the colorectal and 2,303 in the gynecology study). The QoR-15I was completed by 4,791 patients, with a recruitment rate of 92%. Of the completed questionnaires, 742 were filled at a time point after surgery that was longer than that scheduled. Out of the remaining 4,049 questionnaires, 265 had missing values for one or more items, corresponding to a completion rate of 93.4%. All the 3,784 fully completed cases were included in the validation analyses. The flowchart in the (Supplementary material; Supplementary Figure S1) describes the data stratified by type of surgery.

The demographic and clinical characteristics of the included patients are described in Table 1, overall and by type of surgery (colorectal cancer surgery and hysterectomy).

**Table 1:** Demographics and clinical characteristics of the included patients, overall and by type of surgery (colorectal cancer surgery and hysterectomy).

**Patient characteristics**	**Colorectal cancer surgery (*N* = 1972)**	**Hysterectomy (*N* = 1812)**	**Total (*N* = 3,784)**
	*n* (%)	*n* (%)	*n* (%)
**Gender**			
Male	1,115 (56.5)	_	1,115 (29.5)
Female	857 (43.5)	1812 (100)	2,669 (70.5)
**Age – mean (std)**	*71.1 (10.9)*	*55.4 (11.6)*	*63.6 (13.7)*
18–65 yrs	547 (27.7)	1,419 (78,3)	1966 (52.0)
65+ yrs	1,425 (72.3)	393 (21.7)	1818 (48.0)
**American Society of Anesthesiologists classification**			
I– II	1,136 (57.6)	1,511 (83.5)	2,647 (70.0)
III– IV	835 (42.4)	298 (16.5)	1,133 (30.0)
**Marital Status**			
Married	1,345 (68.2)	1,162 (64.1)	2,507 (66.3)
Single	563 (28.6)	621 (34.3)	1,184 (31.3)
Missing	64 (3.3)	29 (1.6)	93 (2.4)
**Employment status**			
Working	445 (22.6)	1,019 (56.2)	1,464 (38.7)
Retired	1,267 (64.3)	339 (18.7)	633 (16.3)
Unemployed	202 (10.2)	431 (23.8)	1,606 (41.4)
Missing	58 (2.9)	23 (1.3)	81 (2.1)
**Education**			
Elementary (5 yrs)	542 (27.5)	187 (10.3)	729 (19.3)
Intermediate (8 yrs)	656 (33.3)	588 (32.5)	1,244 (32.9)
Secondary school	510 (25.9)	811 (44.8)	1,321 (34.9)
Degree	113 (5.7)	189 (10.4)	302 (7.8)
Misssing	151 (7.7)	37 (2.0)	188 (5.0)
**Charlson Comorbidity Index**			
0	1,518 (77.0)	1,658 (91.5)	3,176 (88.0)
≥1	454 (23.0)	154 (8.5)	608 (18.0)
**Presence of at least one complication**			
Surgical complications	314 (15.9)	65 (3.6)	379 (10.0)
Medical complications	307 (15.6)	52 (2.9)	359 (9.5)
Total complications	522 (26.5)	113 (6.2)	635 (16.8)
**Admission in Intensive Care Unit (ICU)**			
No	1720 (87.2)	1770 (97.7)	3,490 (92.2)
yes	252 (12.8)	42 (2.3)	294 (7.6)
**ICU days – median: IQRange (Q1;Q3)**	1: (1; 1)	1: (1; 1)	1: (1; 1)
**Length of Hospital stay, days – median: IQRange (Q1;Q3)**	7: (5; 9)	3: (2; 4)	5: (3; 7)
**Type of surgery**			
Open	361 (18.3)	661 (36.5)	1,022 (26.5)
Laparoscopy or robotic	1,611 (81.7)	1,151 (63.5)	2,762 (73.5)

### Factor structure

Table 2 reports the goodness of fit indices for the measurement models examined in the test group. The unidimensional and the theoretical bidimensional factor models showed unsatisfactory values: in all the three solutions (unidimensional, two orthogonal factors and two correlated factors) CFI was lower than 0.90 and both RMSEA and SRMR were greater than 0.08. The two-factor ESEM solution showed satisfactory values for all the fit indices. As reported in appendix (Supplementary Table S2), items loaded, with value >0.30, in only one factor and all secondary loadings were <0.30, with the only exception of the item “moderate pain” that shown poor loadings in both factors. By the inspection of the content of the items, the first factor was named physical QoR because it included most of the items belonging to the theoretical dimension of “physical well-being” and the second was named mental QoR because it included the two items dealing with anxiety and depression and items related to pain and nausea and vomiting. Given the absence of cross-loading, because of the strength of the correlation between the two ESEM factors (0.437) and in light of the literature that uses the QOR-15 scale as a one-dimensional instrument, we chose to estimate a bifactor model with a general factor of quality of recovery and two group factors, corresponding to the physical and mental ESEM factors.

**Table 2:** Goodness-of-fit indices factorial models estimated on the test sample.

**Models**	**Number free parameters**	*χ* ^2**a**^	**DF**	**AIC**	**BIC**	**Goodness-of-fit**		
						**RMSEA**	**CFI**	**SRMR**
**ONE-FACTOR**	50	1233.079	85	121080.43	121357.69	0.084	0.844	0.081
**TWO ORTOGONAL FACTORS**	50	2203.315	85	122441.44	122718.70	0.115	0.711	0.196
**TWO CORRELATED FACTORS**	51	1220.730	84	121057.41	121340.23	0.085	0.845	0.082
**ESEM**	64	598.872	71	120222.26	120577.17	0.063	0.928	0.043
**BIFCTOR**	65	386.182	70	119901.87	120262.32	0.049	0.957	0.036

CFI, comparative fit index; df, degrees of freedom; AIC, Akaike Information Criteria; BIC Bayesian Information Criteria; RMSEA, root mean square error of approximation; SRMR, standardized root mean square residual; ESEM, exploratory structural equation modeling. ^a^*χ*^2^ value of ps are all < 0.001.

The bifactor model calculated starting from the ESEM solution showed also good fit (RMSEA = 0.049, CFI = 0.957, SRMR = 0.036), returning one general recovery factor and two specific factors of physical and mental recovery. The chi-square difference test comparing the bifactor solution and the nested ESEM solution was statistically significant (Chi-square difference (1) = 55,32, *p* < 0.001), meaning that the bifactor model fits better and is preferable.

Standardized factor loadings for the bifactor model are shown in Table 3 and depicted in Supplementary Figure S2. All items loaded acceptable on the general recovery factor (loading ≥0.30), with the only exception of two items belonging to the mental factor (Item 12. ‘*Severe pain’* and Item 13. *‘Nausea or vomiting’*). Loadings on the group factors (physical and mental recovery) were all <0.30, with the exception of three items for physical factor, while for mental factor all items were > =0.30 with the exception of one items. The ECV value was 0.84 (meaning that 84% of the common variance was due to the general recovery factor), the ω_h_ value for the general factor was 0.70 indicating that the data were sufficiently “one-dimensional”. The ω_s_ values for the physical and mental recovery factors were, respectively, 0.01 and 0.13, indicating that the reliability of the group factors was very low and the calculation of the total score of the questionnaire was recommended. These results support essential unidimensionality of the QoR-15I.

**Table 3:** Standardized bifactor model loadings estimated on the test sample (*N* = 1892).

**QOR-15 items**	**General factor**	**Physical**	**Mental**
1. Able to breathe easily	**0.49**	**0.48**	
2. Been able to enjoy food	**0.62**	0.10	
3. Feeling rested	**0.63**	0.08	
4. Have had a good sleep	**0.47**	0.09	
5. Able to look after personal toilet and hygiene unaided	**0.57**	0.03	
6. Able to communicate with family or friends	**0.51**	**0.42**	
7. Getting support from hospital doctors and nurses	**0.41**	**0.37**	
8. Able to return to work or usual home activities	**0.69**	−0.20	
9. Feeling comfortable and in control	**0.72**	−0.05	
10. Having a feeling of general well-being	**0.87**	−0.20	
11. Moderate pain	**0.30**		0.25
12. Severe pain	0.25		**0.57**
13. Nausea or vomiting	0.20		**0.67**
14. Feeling worried or anxious	**0.30**		**0.52**
15. Feeling sad or depressed	**0.30**		**0.56**

Bold values have a loading ≥ 0.30 on general and group factors.

#### Measurement invariance

The bifactor model showed satisfactory fit measures also in the validation set (RMSEA = 0.052, CFI = 0.951, SRMR = 0.033). To assess the measurement invariance of the QoR-15I between the two types of surgery, we preliminarily examined the goodness of fit of the model in each group of patients, obtaining satisfactory results in both groups (Table 4, M1 and M2). The multi-group analysis supported configural invariance (Table 4, M3: RMSEA = 0.053, CFI = 0.950, SRMR = 0.035) and metric invariance, that is, the loadings were the same in both samples (Table 4, M4: ΔRMSEA = -0.004; ΔCFI = -0.002; ΔSRMR = 0.014). Item intercepts (scalar invariance) were found to be partial invariant across groups: the equality constrain had to be removed from one item (Item 15:*‘Feeling sad or depressed’*), to obtain acceptable fit measures (Table 4, M5: ΔRMSEA = 0.002; ΔCFI = 0.007; ΔSRMR = 0.004).

**Table 4:** Goodness-of-fit indices for bifactor model and measurement invariance across type of surgery of the QOR-15I on the validation set (*N* = 1892).

**Models**		**Number free parameters**			**Goodness-of-fit**			**Comparison**			
	***Multigroup invariance***		*χ* ^2**a**^	**DF**	**RMSEA**	**CFI**	**SRMR**		**ΔRMSEA**	**ΔCFI**	**ΔSRMR**
M1	Colon resection (*N* = 986)	65	240.90	70	0.050	0.957	0.036				
M2	Hysterctomy (*N* = 906)	65	280.57	70	0.058	0.940	0.0035				
M3	Configural invariance	130	518.35	140	0.053	0.950	0.035				
M4	Metric invariance	100	561.28	170	0.049	0.948	0.049	M4 vs. M3	−0.004	−0.002	0.014
M5	Scalar invariance	88	655.60	182	0.052	0.937	0.055	M5 vs. M4	0.003	−0.011	0.006
M6	Partial scalar invariance*	89	623.49	181	0.051	0.941	0.053	M6 vs. M4	0.002	0.007	0.004

*Item 15 intercept freed to be non-invariant across groups.

#### Reliability and validity

To assess the psychometric proprieties of the Italian version of the QoR-15, the whole sample (N = 3,784) was considered. Standardized bifactor loadings on the general as well as on the physical and mental recovery on the total sample were reported in (Supplementary Table S3; Supplementary material).

The overall mean QoR-15I score was 110.3 (SD =21.8), and although the distribution was slightly negatively skewed, skewness and kurtosis coefficient (−0.4 and-0.2, respectively) were consistent with a normal distribution (Supplementary Figure S3 in Supplementary material). Only 18 patients (0.5%) had a QoR-15I score of less than 50 and none of them had the lowest value on the scale, while 123 patients (3.2%) had a score greater than 145 (47 patients – 1.24% – had the highest value), excluding both ceiling and floor effects.

Cronbach’s α and ω_t_ were good (0.85 and 0.84 respectively). Each item was internally consistent (split-half correlation = 0.83) and well correlated with the QoR-15I total score, with values ranging from 0.38 to 0.74.

Construct validity was tested by comparing patients’ QoR-15I scores with length of stay (r = −0.25, 95% CI: −0.28 to-0.22, *p* < 0.001), and occurrence or not of postoperative complications (99.8 ± 22.3 vs. 112.4 ± 21.1) resulting in a standardized mean difference g-Hedge =0.59. These results were consistent for both medical and surgical complications. Duration of surgery does not seem to be related to QoR-15I score (r = −0.09, 95% CI: −0.12 to-0.06, p < 0.001). Higher ASA scores (III-IV vs. I-II), Charlson comorbidity index (1+ vs. 0), and open surgical approach (mini-invasive) showed lower QoR-15I mean scores (108.3 vs. 111.1, g-Hedge =0.13; 107.9 vs. 110.7, g-Hedge =0.14; and 104.1 vs. 112.5, g-Hedge =0.38, respectively). Lower recovery scores were also observed in more complex interventions, as shown by the association with stomas performance in colorectal cancer patients and with malignant cancer in patients undergoing hysterectomy. A strong positive correlation was observed between general VAS health status and QoR-15I score (r = 0.62, 95% CI: 0.60–0.64, *p* < 0.001). In contrast, the correlation with age was not statistically significant (r = −0.02, 95% CI: −0.05 to 0.001, *p* = 0.187) (Table 5). For colorectal cancer, women had lower QoR-15I values (107.2 vs. 110.6, *p* < 0.0005). All of these results were consistent for both types of surgery, except for the role of the Charlson comorbidity index, which was not significantly associated with QoR-15I score in women who underwent hysterectomy.

**Table 5:** QoR-15I scores for different patients’ characteristics, overall and by type of surgery (colorectal cancer surgery and hysterectomy) *N* = 3,784.

**Variables**	**Colorectal surgery**		**Hysterectomy**		**Total patients**	
	**Statistics**	***p*-Value**	**Statistics**	***p*-Value**	**Statistics**	***p*-Value**
Age*	−0.04	0.05	0.07	<0.001	−0.02	0.174
Length of stay*	−0.24	<0.001	−0.27	<0.001	−0.22	<0.001
Medical CC absent	111.0	<0.001	112.0	<0.001	111.5	<0.001
Medical CC present	99.4		97.8		99.1	
Surgical CC absent	111.1	<0.001	111.9	<0.001	111.50	<0.001
Surgical CC present	98.3		100.6		98.6	
Total CC absent	112.5	<0.001	112.3	<0.001	112.4	<0.001
Total CC present	99.9		99.3		99.8	
Well-being VAS*	0.66	<0.001	0.59	<0.001	0.63	<0.001
Surgery duration*	−0.06	0.02	−0.09	<0.001	−0.10	<0.001
Stomia absent Stomia present	110.2 105.3	<0.001				
Malignant neoplasm			110.7	0.03		
Benign pathology			113.1			
ASA I-II	110.3	<0.001	111.8	<0.001	111.1	<0.001
ASA III-IV	107.6		110.4		108.3	
Charlson index 0	109.7	0.04	111.7	0.28	110.7	0.003
Charlson index ≥1	107.2		109.7		107.9	
Open Laparoscopy or robotic	102.2 110.7	<0.001	105.6 114.9	<0.001	104.4112.5	<0.001

Results are reported as mean and mean difference t test.

*Pearson correlation coefficients.

ASA, American Society of Anaesthesiologists; VAS, Visual Analogue Scale; CC, Complications.

## Discussion

In this study, we translated and validated the Italian version of the QoR-15. The QoR-15I proved to be valid and reliable in measuring the quality of recovery after surgery in a sample of patients undergoing elective colorectal cancer resection and in a sample of women hospitalized for elective hysterectomy for cervical or endometrial cancer or for benign uterine disease. The results of the confirmatory factor analyses support the essential unidimensionality of the QoR-15I, although a trace of the multidimensionality of the QoR-40, from which it was derived, remains. The presence of two possible subscales has already been suggested in the work of Stark and colleagues, in which the five dimensions of the QoR-40 were combined into two summary measures, physical and mental well-being (Stark et al., 2013). Although Stark and colleagues identified two domains of recovery, they calculated only one QoR-15 total score. Subsequent studies that attempted to examine the factor structure of the QoR-15 (Kleif et al., 2018; Myles, 2021) applied the bifactor model because it allows for the maintenance of a unidimensional structure while accounting for important factors to group the items (Reise et al., 2013). Our study shows that 84% of the common variance is due to the general recovery factor and that the QoR-15I is sufficiently ‘unidimensional’ and has adequate reliability (ω_h_ = 0.70). Moreover, the ω_s_ values for the physical and mental recovery factors were 0.01 and 0.13, respectively, indicating that the reliability of the group factors is very low and the calculation of the total score of the questionnaire is recommended. Even if the loadings of the items related to the mental specific factor are all around the threshold of 0.30, with a couple of them below, we decided to retain all items and reserve the decision to delete or revise them for future studies.

The items included in the two group factors do not completely overlap with those identified in the original study, which were selected on the basis of their correlation with the QoR. In our study, the mental recovery dimension included also items related to pain and nausea and vomiting. This finding seems to be consistent with the literature, in which several studies evidenced a strong correlation between pain and depression, leading to impaired functioning, lower response to treatment, and limited treatment options (Ghoneim and O’Hara, 2016). In addition, postoperative nausea and vomiting can be influenced by various stimuli, including anxiety, and both appear to originate in the cerebral cortex (Whelan et al., 2012).

Our study also supports the partial invariance of QoR-15I between two different types of patients with very different diagnoses, surgical treatments, and hospital units. To our knowledge, this is the first study to deal with factor structure using a rigorous approach and the first to test its invariance in subgroups of patients with different complex surgeries and different diagnoses. The results show that the QoR-15I is a valid instrument for measuring the construct of recovery after surgery and that comparison of the underlying latent construct between patient groups suggests that they are generally similar for colorectal and gynecologic patients. We conclude that the QoR-15I is appropriate for the subgroups of patients studied here and for future comparisons of mean group differences.

Construct validity was supported by a negative association of QoR-15I scores with all variables related to worse patient condition and more complex surgery: higher ASA score, presence of comorbidities and type of surgery, occurrence of complications, and length of stay. These results were consistent in both patient groups. The performance of a stoma in colorectal cancer patients and a diagnosis of malignancy in hysterectomy patients, which are considered proxies for a more severe condition, were associated with lower QoR scores. In contrast, QoR scores were positively correlated with higher VAS scores. In the colorectal study, we found higher QoR scores in men. These results were comparable to those reported in the original validation work by Stark et al. (2013). According to other studies (Stark et al., 2013; Sá et al., 2015; Lyckner et al., 2018), there was no difference in QoR related to age.

The QoR-15I also proved to be a reliable instrument for assessing quality of recovery, with good internal consistency as measured by a Cronbach’s α of 0.85 and ω_t_ of 0.84 (Cohen, 1988; Reise et al., 2013). Consistent with previous studies (Stark et al., 2013; Kleif et al., 2015; Lyckner et al., 2018; Lee et al., 2021), ceiling and floor effects were not observed with the QoR-15I, allowing good discrimination of patients with different levels of recovery. The successful completion rate (92%) suggests good feasibility. Data reported in the literature on the feasibility of the QoR-15 are heterogeneous (Stark et al., 2013; Kleif et al., 2015; Lyckner et al., 2018) and come from validation studies with a different study design than that of our large pragmatic study.

Several other studies have translated and validated the QoR-15 in other languages (Kleif et al., 2015; Sá et al., 2015; Bu et al., 2016; Lyckner et al., 2018; Demumieux et al., 2020; Kim et al., 2020; Lee et al., 2021). All have found the translated instrument to be valid and reliable in measuring quality of recovery after surgery. A previous Italian translation and validation of the QoR-15 was proposed by Picconi et al. in 2020 (Picconi et al., 2020). Since it had not been published at the start of our clinical trial, we independently translated and validated the English version. In any case, the two translations are very similar, except for the two cultural adaptations we made.

The present study is subject to some limitations. The two trials, having a pragmatic design, involved all regional centers regardless of their expertise in conducting clinical trials and collecting data on patients reported outcomes. In fact, the response rate varied between centers. Although, the 92% recruitment rate was a valuable finding, among non-respondents we found a population that was slightly older and had a more severe profile (longer duration of surgery, longer length of stay, more frequent complications, and access to the intensive care unit), suggesting a selection of respondents with a more favorable recovery profile. Another limitation of the study is the lack of a two-stage administration to calculate the reliability of the test–retest measurement. Even if a baseline measurement is important for a complete validation, we chose to minimize the level of engagement required by the centers for the same reasons we mentioned earlier in relation to the pragmatic design.

Despite the limitations due to the pragmatic design, the involvement of a large regional network of hospitals has allowed us to collect a large sample of population-level data on two different clinical conditions, with the opportunity to examine the factorial structure and measurement invariance of the QoR-15I.

## Conclusion

In sum, this study supports the use of the QoR-15I as a valid, reliable, and clinically feasible instrument for measuring the quality of recovery after surgery. The results of the confirmatory factor analyses suggest that a unique recovery score can be calculated. We further evidenced that this structure is invariant according to the type of surgery, suggesting that the questionnaire has the same meaning and measurement parameters in colorectal and gynecologic patients. These findings support the use of the questionnaire in different contest. Nevertheless, it should be noted that it would be of interest for future studies to test the measurement invariance of the QoR15 in other groups such as men and women or different age groups and to assess whether some items could be removed or revised from the questionnaire.

## Author’s note

Information on the ERAS colon-rectum Piemonte study group members and ERAS Gyneco Piemonte study group members can be found in in Supplementary Datasheet 2.

## Data availability statement

The raw data supporting the conclusions of this article will be made available by the authors, without undue reservation.

## Ethics statement

Study protocols were approved by the Ethical Committees of all participating units. The patients/participants provided their written informed consent to participate in this study.

## Author contributions

EPa, RR, FB, MC, AP, and GC: study design. ERAS Colon‐Rectum Piemonte study group and ERAS Gyneco Piemonte group: Patients’ recruitment and data collection. EPa, RR, ST, and GC: statistical analysis. EPa, RR, and GC: drafting of paper. EPa, RR, GC, VP, FB, MC, AP, ST, EDP, LP, EPi, AR, MR, MM, and MA: revising of paper. All authors contributed to the article and approved the submitted version.

## Funding

This work was supported by the Italian Ministry of Health and the Regione Piemonte as part of the Easy-Net Project, grant number NET-2016-02364191.

## Conflict of interest

The authors declare that the research was conducted in the absence of any commercial or financial relationships that could be construed as a potential conflict of interest.

## Publisher’s note

All claims expressed in this article are solely those of the authors and do not necessarily represent those of their affiliated organizations, or those of the publisher, the editors and the reviewers. Any product that may be evaluated in this article, or claim that may be made by its manufacturer, is not guaranteed or endorsed by the publisher.

## Footnotes

^1^ https://easy-net.info/
