# Supplementary material for: Factor structure of post-operative quality of recovery questionnaire (QoR-15): An Italian adaptation and validation
Source: Front Psychol. 2023 Feb 1;13:1096579. doi: 10.3389/fpsyg.2022.1096579 (PMC9936892; doi:10.3389/fpsyg.2022.1096579)
Supplement: Supplementary file 1 [file Data_Sheet_1.docx]

**Supplementary material**

**Figure S1**. Flow chart of the patients included in theQOR-15I validation analyses.


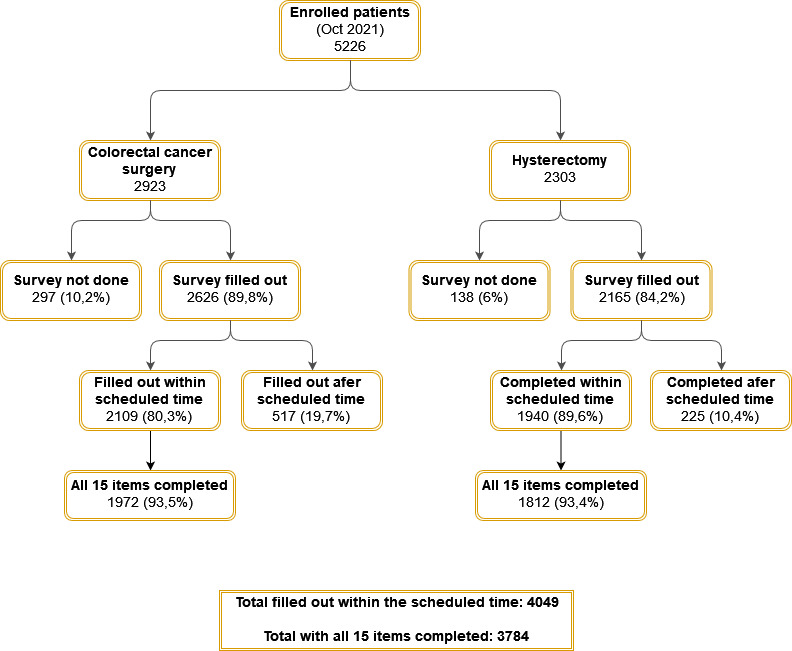


**Figure S2.** Final estimated CFA model (standardized coefficients).

**Figure S3.** QoR-15I scores distribution in the overall sample.

**Table S1.** Italian translated version of the QoR-15 questionnaire.

| **** | **** |
| --- | --- |

**Table S2.** Standardized f**actor loadings estimated with ESEM on the test sample**

|  | **Theoretical structure** | **ESEM solution** | |
| --- | --- | --- | --- |
| **QOR-15 items** |  | **Physical** | **Mental** |
| 1. Able to breathe easily | **Physical** | **0.42** | 0.10 |
| 2. Been able to enjoy food | **Physical** | **0.60** | 0.06 |
| 3. Feeling rested | **Physical** | **0.59** | 0.10 |
| 4. Have had a good sleep | **Physical** | **0.41** | 0.15 |
| 5. Able to look after personal toilet and hygiene unaided | **Physical** | **0.57** | 0.01 |
| 6. Able to communicate with family or friends | **Mental** | **0.45** | 0.08 |
| 7. Getting support from hospital doctors and nurses | **Mental** | **0.36** | 0.06 |
| 8. Able to return to work or usual home activities | **Physical** | **0.76** | -0.14 |
| 9. Feeling comfortable and in control | **Mental** | **0.76** | -0.09 |
| 10. Having a feeling of general well-being | **Mental** | **0.88** | -0.05 |
| 11. Moderate pain | **Physical** | 0.21 | 0.24 |
| 12. Severe pain | **Physical** | -0.01 | **0.62** |
| 13. Nausea or vomiting | **Physical** | -0.11 | **0.74** |
| 14. Feeling worried or anxious | **Mental** | 0.08 | **0.56** |
| 15. Feeling sad or depressed | **Mental** | 0.05 | **0.62** |
| Factors correlation |  | **0.44** | |

Note: Bold values have a loading ≥0.30 into the first or second factor.

**Table S3.** Standardized bifactor model loadings on the total sample N=3784

| **QOR-15 items** | **General factor** | **Physical** | **Mental** |
| --- | --- | --- | --- |
| 1. Able to breathe easily | **0.41** | **0.49** |  |
| 2. Been able to enjoy food | **0.65** | 0.04 |  |
| 3. Feeling rested | **0.65** | 0.11 |  |
| 4. Have had a good sleep | **0.53** | 0.03 |  |
| 5. Able to look after personal toilet and hygiene unaided | **0.58** | 0.08 |  |
| 6. Able to communicate with family or friends | **0.46** | **0.56** |  |
| 7. Getting support from hospital doctors and nurses | **0.30** | **0.50** |  |
| 8. Able to return to work or usual home activities | **0.74** | -0.09 |  |
| 9. Feeling comfortable and in control | **0.71** | 0.18 |  |
| 10. Having a feeling of general well-being | **0.89** | -0.05 |  |
| 11. Moderate pain | **0.34** |  | 0.18 |
| 12. Severe pain | **0.30** |  | **0.50** |
| 13. Nausea or vomiting | 0.27 |  | **0.60** |
| 14. Feeling worried or anxious | **0.34** |  | **0.50** |
| 15. Feeling sad or depressed | **0.31** |  | **0.48** |
