# Supplementary material for: Factor structure of post-operative quality of recovery questionnaire (QoR-15): An Italian adaptation and validation
Source: Front Psychol. 2023 Feb 1;13:1096579. doi: 10.3389/fpsyg.2022.1096579 (PMC9936892; doi:10.3389/fpsyg.2022.1096579)
Supplement: Supplementary file 2 [file Data_Sheet_2.docx]

**ERAS colon-rectum Piemonte study group members**

**Site investigators**

AO Alessandria: Federica Borromeo, Fabio Priora; AO Maggiore della Carità, Novara: Sergio Gentilli, Luca Portigliotti; AO Mauriziano, Torino: Paolo Massucco, Marco Palisi; AO S.Croce e Carle, Cuneo: Maria Carmela Giuffrida; AO San Luigi Gonzaga, Orbassano (TO): Maurizio De Giuli, Aridai Resendiz; Gradenigo – Humanitas, Torino: Paola Bellomo, Silvia Marola; IRCCS Candiolo (TO): Felice Borghi, Luca Pellegrino, Alfredo Mellano, Dario Ribero; Nuovo Ospedale degli Infermi, Biella: Roberto Polastri; Ospedale Civile Agnelli, Pinerolo (TO): Andrea Muratore, Nicoletta Sveva Pipitone; Ospedale degli Infermi, Rivoli (TO): Mauro Garino; Ospedale Cardinal Massaia, Asti: Elisabetta Castagna, Gabriele Pozzo; Ospedale Castelli, Verbania: Andrea Caneparo; Ospedale Civico, Chivasso (TO): Adriana Ginardi, Reggina Lagana; Ospedale Civile, Ciriè (TO): Monica Carrera, Stefania Muzio; Ospedale Civile, Ivrea: Luca Panier Suffat, Ivan Lettini; Ospedale Maggiore, Chieri (TO): Alberto Kiss, Valentina Gentile; Ospedale Martini, Torino: Roberto Saracco, Donatella Scaglione; Ospedale Regina Montis Regalis, Mondovì (CN): Andrea Gattolin, Roberto Rimonda; Ospedale S.Biagio, Domodossola (VCO): Francesco Battafarano, Luigi Oragano; Ospedale S.Croce, Moncalieri (TO): Luca Lorenzin, Carlo Palenzona; Ospedale S.Giacomo, Novi Ligure (AL): Carmine Gianfranco Di Somma, Eliana Giaminardi; Ospedale S.Lazzaro, Alba (CN): Marco Calgaro, Marco Naddeo; Ospedale S.Lorenzo, Carmagnola (TO): Piero Cumbo, Emma Marchigiano; Ospedale S.Spirito, Casale M.to (AL): Francesca Cravero, Marco Amisano, Francesco Lemut; Ospedale San Giovanni Bosco, Torino: Tiziana Viora, Luciano Bonaccorsi; Ospedale Sant’Andrea, Vercelli: Silvio Testa, Clemente De Rosa; Ospedale SS.Annunziata, Savigliano (CN): Marco Brunetti; Ospedale SS.Trinità, Borgomanero (NO): Matteo Gatti, Presidio Cottolengo, Torino: Carlo Bima, Enrico Gibin; Ospedale Maria Vittoria, Torino: Francesco Quaglino, Federico Festa, Luca Bonatti; AOU Città della Salute e della Scienza, Torino: Mario Morino, Marco Ettore Allaix, Paolo De Paolis, Ida Marina Raciti, Mauro Santarelli, Gitana Scozzari.

**Study coordination**

AO S.Croce e Carle, Cuneo: Danilo Donati, Maurizio Meineri, Sarah Palmisano; IRCCS Candiolo: Felice Borghi, Luca Pellegrino; AOU Città della Salute e della Scienza, Torino: Giovannino Ciccone, Rosalba Galletti, Eva Pagano, Sergio Sandrucci; AO Mauriziano, Torino: Ilaria Bachini, Anna De Magistris, Barbara Mitola, Paolo Massucco, Alessio Rizzo; AO San Luigi Gonzaga, Orbassano and Università degli Studi di Torino (TO): Pietro Caironi; Humanitas, Torino: Monica Rolfo; Regione Piemonte: Anna Orlando; Rete Oncologica Piemonte e Valle d’Aosta: Oscar Bertetto, Massimo Aglietta, Mario Airoldi, Alessandro Comandone.

**Technical staff**

AOU Città della Salute e della Scienza, Torino: Francesco Brunetti, Corinna Defilè, Vitor Hugo Martins, Lisa Giacometti, Matteo Papurello, Fabio Saccona, Danila Turco.

**ERAS Gyneco Piemonte study group members**

**Site investigators**

AO Alessandria: Vittorio Aguggia, Federica Borromeo, Stefano Prigione; AO Maggiore della Carità, Novara: Katia Schipani, Daniela Surico; AO Mauriziano, Torino: Enrico Badellino, Annamaria Ferrero; AO S.Croce e Carle, Cuneo: Barbara Franzoso, Elisa Peano, Andrea Puppo; IRCCS Candiolo (TO): Francesco Marocco; Nuovo Ospedale degli Infermi, Biella: Stefano Uccella, Chiara Violino; Ospedale Civile Agnelli, Pinerolo (TO): Marco Canestrelli, Daniela Dompè; Ospedale degli Infermi, Rivoli (TO): Gabriele Molina; Ospedale Cardinal Massaia, Asti: Carlo Bocci, Paola Ferraris; Ospedale Castelli, Verbania: Riccardo Fiorentino; Ospedale Civico, Chivasso (TO): Maurizio Brusati; Ospedale Civile, Ciriè (TO): Antonio Alfeo, Mario Gallo, Romeo Geranio; Ospedale Civile, Ivrea: Fabrizio Bogliatto; Ospedale Maggiore, Chieri (TO): Massimo Mosetti, Giacomo Vaudano; Ospedale Martini, Torino: Marco Camanni, Elena Del Piano; Ospedale Regina Montis Regalis, Mondovì (CN): Alice Peroglio Carus, Elisa Piovano, Marta Sciandra; Ospedale S.Croce, Moncalieri (TO): Pier Luigi Montironi, Andrea Scoletta; Ospedale S.Giacomo, Novi Ligure (AL): Federico Tuo,; Ospedale S.Lazzaro, Alba (CN): Enrica Bar, Alessandro Antonio Buda; Ospedale S.Spirito, Casale M.to (AL): Stefania Cigna Zorzetti, Francesco Lemut; Ospedale SS.Annunziata, Savigliano (CN): Alessio Garetto, Monica Mascher; Ospedale SS. Pietro e Paolo, Borgosesia (VC): Enrico Negrone, Elvira Sorbilli; Ospedale SS.Trinità, Borgomanero (NO): Roberto Biggiogera; AOU Città della Salute e della Scienza, Torino: Donato Mastrantuono, Claudio Plazzotta, Paolo Zola.

**Study coordination**

AO S.Croce e Carle, Cuneo: Andrea Puppo; Ospedale Martini, Torino: Marco Camanni, Elena Del Piano; AOU Città della Salute e della Scienza, Torino: Giovannino Ciccone, Eva Pagano, Paolo Zola; Ospedale Regina Montis Regalis, Mondovì (CN): Elisa Piovano; AO Mauriziano, Torino: Paola Coata, Anna De Magistris, Barbara Mitola, Alessio Rizzo; Humanitas, Torino: Monica Rolfo, Laura Ceretto Giannone, Daria Bongiovanni; Ospedale S.Croce, Moncalieri (TO): Andrea Scoletta; Regione Piemonte: Anna Orlando; Rete Oncologica Piemonte e Valle d’Aosta: Oscar Bertetto, Massimo Aglietta, Mario Airoldi, Alessandro Comandone.

**Technical staff**

AOU Città della Salute e della Scienza, Torino: Francesco Brunetti, Corinna Defilè, Vitor Hugo Martins, Lisa Giacometti, Matteo Papurello, Fabio Saccona, Danila Turco.
